# Purinergic Receptors in Ocular Inflammation

**DOI:** 10.1155/2014/320906

**Published:** 2014-07-14

**Authors:** Ana Guzman-Aranguez, Xavier Gasull, Yolanda Diebold, Jesús Pintor

**Affiliations:** ^1^Department of Biochemistry and Molecular Biology IV, Faculty of Optics and Optometry, Universidad Complutense de Madrid, C/Arcos de Jalón 118, 28037 Madrid, Spain; ^2^Spanish Cooperative Thematic Research Network in Ocular Prevalent and Chronic Pathology (RETIC), Instituto de Salud Carlos III, Madrid, Spain; ^3^Neurophysiology Lab, Department of Physiological Sciences I, Medical School, Universitat de Barcelona, Barcelona, Spain; ^4^Biomedical Research Institute August Pi i Sunyer (IDIBAPS), Barcelona, Spain; ^5^Ocular Surface Group, Institute for Applied Ophthalmobiology (IOBA), University of Valladolid, Valladolid, Spain; ^6^Biomedical Research Networking Center on Bioengineering, Biomaterials and Nanomedicine (CIBER-BBN), Spain

## Abstract

Inflammation is a complex process that implies the interaction between cells and molecular mediators, which, when not properly “tuned,” can lead to disease. When inflammation affects the eye, it can produce severe disorders affecting the superficial and internal parts of the visual organ. The nucleoside adenosine and nucleotides including adenine mononucleotides like ADP and ATP and dinucleotides such as P^1^,P^4^-diadenosine tetraphosphate (Ap_4_A), and P^1^,P^5^-diadenosine pentaphosphate (Ap_5_A) are present in different ocular locations and therefore they may contribute/modulate inflammatory processes. Adenosine receptors, in particular A_2A_ adenosine receptors, present anti-inflammatory action in acute and chronic retinal inflammation. Regarding the A_3_ receptor, selective agonists like N^6^-(3-iodobenzyl)-5′-N-methylcarboxamidoadenosine (CF101) have been used for the treatment of inflammatory ophthalmic diseases such as dry eye and uveoretinitis. Sideways, diverse stimuli (sensory stimulation, large intraocular pressure increases) can produce a release of ATP from ocular sensory innervation or after injury to ocular tissues. Then, ATP will activate purinergic P2 receptors present in sensory nerve endings, the iris, the ciliary body, or other tissues surrounding the anterior chamber of the eye to produce uveitis/endophthalmitis. In summary, adenosine and nucleotides can activate receptors in ocular structures susceptible to suffer from inflammatory processes. This involvement suggests the possible use of purinergic agonists and antagonists as therapeutic targets for ocular inflammation.

## 1. Introduction

Inflammation is the biological process triggered when a vascular tissue needs to be repaired from an injury or faces a microbial challenge. This process is normally self-limited; however, in the absence of a proper return to homeostasis it can turn into a damaging condition for tissues [1, 2]. It works throughout complex and specific interactions between cells and mediator molecules in the damaged tissue that require a fine and well-tuned molecular regulation. In the absence of a prompt resolution of the acute inflammatory response, affected tissue may progress to a chronic inflammatory status that leads to disease [3].

As many organs, human eye suffers important and sometimes devastating inflammatory diseases. In such cases, inflammation can be either cause or consequence of disorders affecting different structures in the anterior part of the eye, the intraocular compartment, or both. Examples of such conditions include lacrimal keratoconjunctivitis, severe allergic diseases, severe cicatrizing conjunctivitis, uveitis (intraocular inflammation of the uvea, retina, vitreous body, and/or optic nerve head), age-related macular degeneration, diabetic retinopathy, and its major complication, and proliferative vitreoretinopathy. Also, it is important to note that ocular infections, traumas, and surgery also involve inflammatory processes that can lead to a vision-threatening situation if not properly controlled. Many efforts have been invested in the development of therapeutic strategies to confront such diseases (for recent reviews see [4–6]), even using the aid of novel technology-related strategies, such as drug delivery systems or gene therapy (for recent reviews see [7, 8]). However, a much deeper knowledge of molecular pathophysiology mechanisms and mediators underlying ocular inflammation-related disorders is necessary.

The eye has its own mechanism to protect itself from inflammation, which is the so called immune privilege [9]. The biological significance of such “privilege” is the active tolerance to foreign antigens exerted by the ocular immune system. In addition, the tightly sealed blood-ocular barriers prevent the passage of inflammatory cells and molecules from the blood into the eye. Altogether, the collateral inflammation that is associated with the normal immune response is avoided. However, the immune privilege can fail and inflammation can eventually develop with the involvement of different mediators.

There are several families of molecules that have been described as active participants in ocular inflammatory diseases. These include immune mediators such as cytokines and chemokines and enzymes such as matrix metalloproteinases, lipids, growth factors and their receptors, and neurotransmitters and their receptors. These last ones are especially interesting because they are involved in maintaining the immune privilege [10], among other physiological activities. Different neuropeptides and their receptors have been identified in ocular structures, including the cornea, sclera, iris, ciliary body, ciliary process, and the retina [11]. In addition, there are examples of the involvement of not only neuropeptides, but also biogenic amines, amino acids, acetylcholine, or purines, in physiological processes of the eye that can be affected by inflammation. Thus, the knowledge of the clinical impact that neuropeptides and their receptors may have is growing in parallel with their envisioned therapeutical applications in ocular inflammatory diseases.

The role of parasympathetic and sympathetic nerves in conjunctival goblet cell functioning has been reported (for review see [12]). The parasympathetic nerves contain the neurotransmitters acetylcholine and vasoactive intestinal peptide (VIP), and the sympathetic nerves contain norepinephrine and neuropeptide Y. Also, purinergic receptor P2Y_2_ agonists, such as UTP and ATP, are capable of stimulating both goblet cell mucin secretion and stratified squamous cell fluid (water and electrolytes) secretion to tear film [12]. Sensory nerves of cornea and conjunctiva contain neurotransmitters such as substance P, calcitonin gene-related peptide, and galanin, which can activate a neural reflex to stimulate conjunctival goblet cell secretion [13]. The reduction in corneal sensation that takes place in chronic inflammatory disease of the ocular surface can impair the secretion of mucin and water components of the tear film.

Histamine produced by conjunctival sensitized mast cells is a well-known mediator of the allergic pathology affecting the eye [14]. It is secreted to conjunctival tissues and the tear film, along with other mast cell-derived irritant mediators, after the allergen challenge and triggers different allergic inflammation-related processes [15]. It not only increases vasodilation and vascular permeability to immune cells, but also directly acts upon specific receptors present in conjunctival epithelial cells to stimulate goblet cell mucin secretion [16]. Besides, histamine exerts a chemotactic effect on various immune cell types through a complex cytokine network thus amplifying its biological activities.

The eye has a small piece of brain in it, that is, the retina. It is long known the importance of neurotransmitters and their receptors for the processing of visual information within the retinal tissue [17]. Catecholamine neurotransmitters, mainly dopamine, are key for retinal neuron functioning in the vertebrate retina. Also, the role exerted by excitatory and inhibitory amino acids as well as acetylcholine in the visual process is well established [18]. Glutamate, aspartate, gamma-aminobutyric acid, taurine, and glycine are normal neurotransmitter or neuromodulatory agents for photoreceptors and other retinal neurons, such as horizontal and amacrine cells. Müller cells, the main glial cell of the retina, also secrete neurotransmitters and express a wide variety of neurotransmitter receptors, reflecting their participation in the physiological signaling between neurons and glial cells [19]. However, glutamate-mediated loss of retinal ganglion cells occurs in glaucoma and retinal vessels occlusion (central and branch retinal artery and retinal vein) [20]. Cooperation between inflammatory cytokines and glutamate receptors has been proposed as one of the mechanisms responsible for a toxic damage on retinal cells related to glutathione depletion [21]. Müller cells protect neurons from glutamate toxicity. It is now widely accepted that almost any retinal degenerative disease is associated with Müller cell gliosis, which is a complex series of functional changes that occurs as a consequence of retinal inflammation accompanying the degenerative process. Gliosis impedes Müller cell protective role against glutamate toxicity and impairs their neurotransmitter recycling activity in the glioneuronal interactions [22].

Among the plethora of transmitters present in the ocular structures, nucleosides and nucleotides emerge as remarkable molecules with the ability to regulate many biochemical and physiopathological processes. Their actions are mediated by membrane receptors termed purinergic receptors that can be divided into adenosine P1 or A receptors and nucleotide receptors named as P2. Adenosine receptors can be divided into A_1_, A_2A_, A_2B_, and A_3_ and these receptors are only sensitive to the nucleoside adenosine. On the contrary, P2 receptors are divided into two main groups, ionotropic P2X and metabotropic P2Y receptors, and are sensitive to adenine, to guanine nucleotides, and also to dinucleotides such as dinucleoside polyphosphates.

The diversity of receptors in the eye structures reflects the importance of these molecules in processes such as tear secretion, intraocular pressure homeostasis, lens accommodation, or retinal functioning. Moreover, use of nucleosides and nucleotides, naturally occurring and synthetic, has been suggested to rescue the eye from some pathological conditions. Indeed there is a chance for the development of patents based on nucleotides as a therapeutic approach since it has been possible to relate nucleoside/nucleotide levels with pathological conditions, such as dry eye or glaucoma, for example. The review of the patent literature did not bring any document related to ocular inflammatory processes. In this sense it might be of interest the development of new inventions for the treatment of ocular inflammation based on purinergic agonists and antagonists.

The scientific literature that studies the relation of purines and the eye have provided a disperse number of papers describing the involvement of these molecules in ocular inflammatory processes. In this sense, the present work reviews and groups the existing works in the field by structuring them in two main groups, on the one hand, actions mediated by means of adenosine receptors and on the other hand those occurring by nucleotide receptors.

## 2. Adenosine Receptors

**Figure 1:**
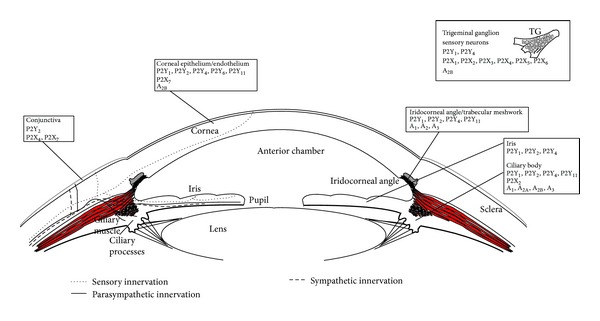
Purinergic receptors identified in the ocular anterior segment. Purinergic receptors localized in the different ocular parts/structures of the ocular anterior segment are shown.

Adenosine is elevated at sites of tissue damage resulting from inflammation or hypoxia [23, 24]. Adenosine can be formed intracellularly and diffuse into the extracellular space via equilibrative nucleoside transporter, or extracellularly from released ATP by ectonucleotidases, CD39 and CD73. Under stress and ischemic conditions, the local tissue concentration of extracellular adenosine is increased due to its synthesis from the released ATP. Adenosine has been proposed to modulate a variety of physiological responses including inflammation and immunity by stimulating specific adenosine receptors (AR) [25, 26]. To date, four adenosine receptor subtypes A_1_, A_2A_, A_2B_, and A_3_ have been identified that belong to the family of seven transmembrane G protein-coupled receptors [27]. The A_1_ and A_3_ adenosine receptors preferentially couple to G_i_ protein to inhibit adenylate cyclase and consequently the production of cyclic AMP (cAMP), and the A_2A_ and A_2B_ subtypes stimulate the production of cAMP by coupling to G_s_. Expression of adenosine receptors has been described in different eye locations (Figure 1). The presence of A_2B_ adenosine receptors [28] has been detected on bovine corneal endothelium. In the ciliary epithelium, A_1_, A_2A_, and A_2B_ adenosine receptor mRNAs were found in the ciliary processes of rat using* in situ* hybridization [29]. Later, A_3_ adenosine receptor mRNA expression was also detected in cultured human ciliary epithelial cells and rabbit ciliary processes by RT-PCR [30]. In the retina, A_2A_ adenosine receptor mRNA expression was mainly found in the inner nuclear layer and ganglion cell layer and to a lesser extent in the outer nuclear layer. Likewise, A_1_ and A_3_ adenosine receptor mRNAs were identified in the ganglion cell layer of the retina [29, 31]. In addition, A_2A_ and A_2B_ adenosine receptors are also present in retinal pigment epithelial cells [29, 32] as well as in Müller cells [33].

### 2.1. A_1_ Adenosine Receptors

Conflicting conclusions about the effect of A_1_ adenosine receptors on inflammation have been reported. Thus, A_1_ adenosine receptor has been implicated as a potent anti-inflammatory mediator in various inflammatory models of several organ systems, including the kidney [34], heart [35], liver [36], and brain [37]. On the contrary, in the lung, pharmacologic blocking of A_1_ adenosine receptors attenuated lipopolysaccharide (LPS)-induced lung injury in cats [38]. Likewise, in an allergic mouse model of asthma, A_1_ adenosine receptors have been shown responsible for altered vascular reactivity, increased airway hyperresponsiveness, and systemic inflammation [39].

In the eye, there is no data about the role of this receptor in ocular inflammation. To date, it has been only showed that A_1_ adenosine receptor mediates IL-6 trophic effect on retinal ganglion cells [40]. IL-6 is a pleiotropic cytokine classically denominated proinflammatory, but, additionally, it has been demonstrated that this cytokine is able to increase the survival of retinal ganglion cells [41]. It remains unknown whether A_1_ adenosine receptor could also take part in some proinflammatory actions induced by this cytokine in the retina apart from the IL-6 trophic effect on retinal ganglion cells.

### 2.2. A_2A_ Adenosine Receptors

**Figure 2:**
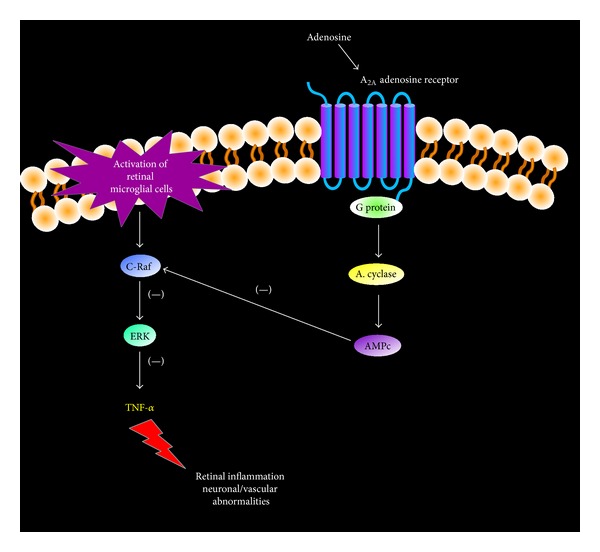
Regulation of retinal inflammation by A_2A_ adenosine receptor. Pathways proposed to be involved in anti-inflammatory effect of A_2A_ adenosine receptor in the retinal microglial cells during pathologies such as diabetes or traumatic optic neuropathy. A_2A_ adenosine receptor activation reduces TNF-*α* release by repressing the inflammatory cascade C-Raf/ERK in activated retinal microglia.

Substantial lines of evidence have suggested that the anti-inflammatory effects of extracellular adenosine are mainly mediated by A_2A_ adenosine receptors [25, 42]. The anti-inflammatory action of A_2A_ adenosine receptors in acute and chronic retinal inflammation has been demonstrated [43, 44]. Using cultured retinal microglia cells activated by LPS as an* in vitro* model of acute neuroinflammation, Liou et al. [44] showed that A_2A_ adenosine receptor activation in the stressed retinal microglial cells efficiently inhibited LPS-induced TNF-*α* release. The protective role of A_2A_ adenosine receptor in chronic retinal inflammation associated to diabetic retinopathy has also been examined [43, 45]. Diabetic retinopathy has been categorized as a vascular-neuroinflammatory disease. Among the early signs of diabetic retinopathy are retinal inflammatory reactions, breakdown of the blood-retinal barrier function, and loss of retinal neurons [46–48]. As the disease progresses, the retina may be damaged by oxidative stress induced by hyperglycemia, or advanced glycation end products [49, 50]. This stress damages vascular and neuronal tissues of the retina and activates microglial cells [51]. Activated microglia further exacerbate the damage by releasing cytotoxic molecules (glutamate, reactive oxygen species) and proinflammatory mediators, such as TNF-*α* [52, 53]. Thus, local inflammation has a relevant contribution in the pathogenesis of diabetic retinopathy. To elucidate the role of A_2A_ adenosine receptor in diabetic retinopathy, the effect of A_2A_ adenosine receptor ablation on diabetic mice was analyzed [43]. Knockout A_2A_ adenosine receptor mice had significantly more retinal terminal deoxynucleotidyl transferase dUTP nick end labeling (TUNEL)-positive cells, TNF-*α* release, and intercellular adhesion molecule 1 (ICAM-1) expression compared with diabetic wild type [43]. Interestingly, together with these changes, an altered microglia phenotype was observed in the knockout A_2A_ adenosine receptor mice. In this sense, in a diabetic milieu, microglia transformed from their ramified resting state into an amoeboid shape, the activated and cytokine-releasing state, and this phenotypic configuration was more evident in the knockout A_2A_ adenosine receptor diabetic mice than in diabetic wild-type [43]. Moreover, treatment of diabetic mice with the A_2A_ adenosine receptor agonist CGS21680 (3-[4-[2-[[6-amino-9-[(2R, 3R, 4S, 5S)-5-(ethylcarbamoyl)-3,4-dihydroxy-oxolan-2-yl]purin-2-yl]amino]ethyl]phenyl]propanoic acid) attenuated the morphological transformation of ramified microglia into an activated ameboid microglia and resulted in marked decreases in diabetes-induced retinal cell death and TNF-*α* release [43]. Inhibition of reactive microglial phenotype acquisition is not the only mechanism by which A_2A_ adenosine receptor regulates inflammation in diabetic retinopathy. Additional studies using microglial retinal cells treated with amadori-glycated albumin (AGA) (a risk factor in diabetic disorders) showed that activation of A_2A_ adenosine receptor attenuated AGA-induced TNF-*α* release by repressing the inflammatory cascade C-Raf/extracellular signal-regulated kinase (ERK) in activated microglia (Figure 2) [43, 45].

Considering these findings about the protective role of A_2A_ adenosine receptor activation in diabetes-induced retinal inflammation, abnormality in adenosine metabolism could have influence on retinal complications in diabetic retinopathy. In this context, an increased expression and activity of catabolic enzyme adenosine deaminase-2 (ADA2), which represent a critical checkpoint in the regulation of extracellular adenosine levels and, consequently, in the control of receptor stimulation and function, have been identified in human and porcine retinas with diabetes as well as in AGA-treated porcine and human microglia cells [54]. Moreover, TNF-*α* release was induced in AGA-treated microglia cells and that TNF-*α* release was blocked by ADA2-neutralizing antibody or ADA2 siRNA [54]. These results confirm that abnormality in adenosine metabolism can contribute to retinal inflammation in diabetic retinopathy and suggest that the anti-inflammatory activity of A_2A_ adenosine receptor signaling can be impaired in diabetic retinopathy due to increased ADA2 activity.

Taking into advance the ability of A_2A_ adenosine receptor to offer protection against retinal inflammation in diabetic retinopathy, the use of the A_2A_ adenosine receptor agonist CGS21680 in other ocular retinal pathologies in which proinflammatory mediators are released has also been examined [55]. The A_2A_ adenosine receptor agonist administration significantly attenuated the expression of inflammatory (TNF-*α*, IL-6, and ICAM-1) and cell death markers in a mouse model of traumatic optic neuropathy (a disease characterized by retinal ganglion cell death, which is closely related to the local production of reactive oxygen species and inflammatory mediators from activated microglial cells) [55]. A_2A_ adenosine receptor agonist anti-inflammatory action was mediated by blocking ERK activation and subsequent cytokine release in traumatic optic neuropathy activated microglia cells (Figure 2).

On the other hand, it has been described the contribution of adenosinergic pathway through A_2A_ adenosine receptor on protective regulatory immunity in a mouse model of human autoimmune uveitis [56]. Thus, A_2A_ adenosine receptor activation on T cells was associated with antigen-presenting cells (APC) induction and activation of Tregs (regulatory T cells), which mediate a postexperimental autoimmune uveoretinitis regulatory immune response to ocular autoantigens protecting from recurrence of uveitis [56].

### 2.3. A_2B_ Adenosine Receptors

Discrepancy between anti-inflammatory and proinflammatory effects has been observed in several tissues for A_2B_ adenosine receptors [57]. This apparent contradiction might be related to differences between the acute and chronic models of inflammation studied, playing the receptor different roles at different points during the progression of inflammation. Furthermore, A_2B_ adenosine receptors may play different roles even in similar types of inflammation but occurring in different tissues [57–59].

Little is known about the role of A_2B_ adenosine receptor in the eye. A gradual increase in A_2B_ adenosine receptor has been reported after alkali burn-induced corneal inflammation and neovascularization. As A_2B_ adenosine receptor was not expressed by normal cornea, it suggests that the A_2B_ adenosine receptor detected after alkali burns was produced in the cornea by infiltrated inflammatory cells [60]. In agreement with this finding, it has been detected that A_2B_ adenosine receptor seems to be mainly expressed in inflammatory cells [61].

### 2.4. A_3_ Adenosine Receptors

The A_3_ adenosine receptor is highly expressed in inflammatory cells whereas low or almost no expression is found in normal cells [62], rendering the A_3_ adenosine receptor as a potential therapeutic target. A_3_ adenosine receptor upregulation can be attributed to several factors, including elevated adenosine and cytokines, which are characteristic of the microenvironment of inflammatory cells [63]. Under these conditions, the binding of adenosine to their cell surface receptors might induce, through an autocrine pathway, the expression of its own receptors [64, 65]. Additionally, it has been proposed that the proinflammatory cytokine TNF-*α* can induce an increase of the phosphatidylinositol 3-kinase (PI3K) and protein kinase B (PKB)/Akt expression levels, resulting in upregulation of cAMP response element-binding (CREB) and nuclear factor-kappaB (NF-*κ*B) which translocate to the nucleus to act as A_3_ adenosine receptor transcription factors [62].

Selective A_3_ adenosine receptor agonists are being developed for the treatment of inflammatory diseases such as rheumatoid arthritis, osteoarthritis, psoriasis, and inflammatory bowel diseases [66]. One of these agonists is the compound CF101 (N^6^-(3-iodobenzyl)-5′-N-methylcarboxamidoadenosine) which exerts a robust anti-inflammatory effect in experimental animal models of inflammatory diseases [67–70]. The mechanism of action mediating the anti-inflammatory effect of CF101 includes downregulation of NF-*κ*B signaling pathway, leading to inhibition of proinflammatory cytokines (TNF-*α*, IL-6, and IL-12), macrophage inflammatory proteins (MIPs-1a, MIP-2), and receptor activator of NF-*κ*B ligand (RANKL), resulting in apoptosis of inflammatory cells [68, 71]. In addition, a direct antiproliferative effect of CF101 towards autoreactive T cells has been observed [72].

The anti-inflammatory effects of CF101 via A_3_ adenosine receptor has prompted to explore its use for the treatment of inflammatory ophthalmic diseases such as dry eye and uveoretinitis. Dry eye syndrome is an inflammatory condition of the eye characterized by a massive production of proinflammatory cytokines [73–75]. Desiccating stress induces tear hyperosmolarity, activating intracellular signaling pathways that initiate the production of proinflammatory cytokines. These inflammatory mediators promote the activation (maturation) of immature APCs and induce their migration to draining lymphoid tissues. The APCs are responsible for priming naive T cells in the lymphoid compartment, leading to the expansion of autoreactive CD4^+^ helper T cell (TH) subtype 1 and T_H_17 cell subsets. T cells subsequently infiltrate the ocular surface, where they secrete additional proinflammatory cytokines [76].

A phase II clinical study (randomized, multicenter, double-masked, placebo-controlled, and parallel group) exploring the effect of CF101 on patients with moderate to severe dry eye syndrome has been performed. CF101 administrated orally (1 mg/day for 12 weeks), induced a statistically significant improvement in the corneal staining and an improvement in the tear break-up time and tear meniscus height in patients with dry eye syndrome [77]. In good agreement with previous trials [78], the drug was very well tolerated and no severe adverse effects were detected. It has been suggested that the improvement in the corneal staining and tear break-up time in the study group might be due to reduced inflammation on the ocular surface following direct interaction between CF101 and its receptors on inflammatory cells [79]. However, additional proofs of reduction of inflammation are required to fully confirm this notion.

An experimental mice model of uveitis has been used to test the anti-inflammatory action of CF101. Oral treatment with CF101 (10 *μ*g/kg, twice daily), initiated upon disease onset, improved uveitis clinical score measured by fundoscopy and ameliorated the pathological manifestations of the disease [72]. A decrease in PI3K and STAT (signal transducer and activator of transcription) protein levels in the lymph nodes of experimental autoimmune uveitis mice was detected upon CF101 treatment. Both proteins are known to be involved in the production of proinflammatory cytokines [80, 81] and, indeed, inhibition of interleukin-2, TNF-*α*, and interferon-*γ* (IFN-*γ*) production was also found in CF101-treated animals [72]. Furthermore, CF101 suppressed the antigen-specific proliferation of autoreactive T cells. Overall these results indicate the marked anti-inflammatory effect of CF101 and support further investigation of this drug for uveitis treatment.

## 3. Ocular Sensory Innervation and Purinergic Receptors P2 Involved in Ocular Inflammation

The trigeminal ganglion through the ophthalmic nerve provides nonvisual sensory innervation of the eye. Sensory neurons innervating the eye detect noxious or potentially noxious stimuli in order to protect the eyeball, elaborate responses to minimize damage, and promote tissue repair. These sensory neurons transduce mechanical, thermal, and chemical stimuli in the noxious range or close to it. Most of the sensory nerve endings innervate the front of the eye, in particular, the cornea and conjunctiva, but important innervation is present in the uvea, where it has a critical role on ocular inflammation [82].

Autonomic parasympathetic innervation of the eye is supplied by the Edinger-Westphal nucleus in the brainstem through the oculomotor nerve [83, 84]. Parasympathetic nerve fibers synapse in the ciliary ganglion and enter the ocular globe through the short ciliary nerves to innervate the iris, the ciliary body and ciliary muscle, and parts of the iridocorneal angle (uveal trabecular meshwork and scleral spur). Some parasympathetic fibers come from the pons through the geniculate ganglion (Petrosal). Later they synapse in the pterygopalatine ganglion before entering the eye [85]. In parallel, sympathetic nerve fibers arise from the superior cervical ganglion and enter the eyeball though the long and short ciliary nerves. They innervate the ciliary body (central stroma and stroma of the ciliary processes), the iris, and parts of the iridocorneal angle. Nonsignificant autonomic innervation is present in the cornea, which is innervated exclusively by sensory fibers.

Different studies have provided evidence for the presence of purinergic receptors in sensory neurons from the trigeminal ganglion (Figure 1). P2X_3_ receptor mRNA and protein are found in the cell bodies of both small and large sensory neurons, which has the highest level of expression among these neurons and, in particular, in peptidergic neurons [86]. In contrast, only a small percentage of IB4-binding neurons express this receptor in trigeminal ganglia. Lower levels are found for P2X_1_, P2X_2_, P2X_4_, P2X_5_, and P2X_6_ [86–88]. mRNA and protein for P2Y_1_ and P2Y_4_ receptors are also present and in many neurons, colocalized with P2X_3_ receptors [89]. Despite the studies in trigeminal ganglion neurons, there is a lack of specific studies on purinergic receptors in the sensory nerve endings innervating the anterior part of the eye (cornea, sclera, and conjunctiva) or the uvea (iris and ciliary body). Although no information is available for ocular nerves, purinergic receptors P2Y_1_, P2Y_2_, P2Y_4_, and P2Y_6_ are present in the corneal epithelium and endothelium cells [90] (Figure 1). In fact, injury to corneal epithelial cells results in nucleotide release and mobilization of a calcium wave from the epithelium to the neurons [91]. It has been hypothesized that ATP is initially released from epithelial cells and then followed by a release of ATP and glutamate from neuronal processes that activate purinergic and N-methyl-D-aspartate (NMDA) receptors, contributing to the wound response [91]. In humans, P2X_7_ receptor mRNA is also found in the cornea and upregulated in diabetic patients. Evidence indicates that corneal epithelial cells express full-length and truncated forms of P2X_7_, allowing P2X_7_ to function as a multifaceted receptor that can mediate cell proliferation and migration or cell death [92].

In parallel, the conjunctiva, the wet mucosal membrane of the eye, is highly exposed to the environment and at the same time very sensitive to the damaging effects of inflammation. The ocular surface therefore requires a carefully balanced mechanism to initiate inflammation only when absolutely necessary. Here, hybridization to P2Y_2_ receptor mRNA has been observed in the palpebral and bulbar conjunctival epithelium, including goblet cells, the corneal epithelium, and in meibomian gland sebaceous and ductal cells [93]. In addition, recent studies [94] have reported that the purinergic receptors P2X_4_ and P2X_7_ and the bacterial Toll-like receptor 2 (TLR2) are present and functional in conjunctival goblet cells and are involved in the priming and activation of the NLRP3 inflammasome, initiated by danger associated molecular patterns (DAMPs) such as ATP. The P2X_7_ receptor-NLRP3 inflammasome complex modulates the release of the inflammatory cytokines IL-1b and IL-18 and it seems to be involved in the primary Sjögren's syndrome pathology in the salivary glands and likely in Sjögren's derived ocular dryness (xerophthalmia) [95].

In the anterior uvea, purinergic receptors P2Y_1_, P2Y_2_, and P2Y_4_ have been found in the iris [90]. The same receptors and P2Y_11_ have also been observed in both layers of the ciliary body epithelium (pigmented and nonpigmented) in the rabbit and monkey eye (Figure 1), in addition to a variety of structures within the choroid [90, 93]. Functional evidence of P2Y_2_ receptor activity has also been reported in these tissues [96, 97]. In turn, ocular ciliary epithelial cells are known to store and release ATP, an endogenous P2Y_2_ receptor agonist, providing a potential source of extracellular nucleotides for autocrine regulation of intraocular pressure [98]. In this sense, ATP it is known to be released from antidromically stimulated trigeminal sensory nerve endings in the ciliary body and, as a consequence, a significant increase of ATP is found in the aqueous humor [99]. This provides evidence that ATP released by ocular sensory innervation or after injury of ocular tissues can activate both sensory nerve endings and purinergic receptors present in the iris, ciliary body, or other tissues surrounding the anterior chamber of the eye to produce uveitis/endophthalmitis. In addition to the cornea and sclera, abundant sensory nerve terminals are present in the iris and anterior uvea, which detect mechanical, thermal, and chemical stimuli, contributing to neurogenic inflammation (inflammation of neural origin) by releasing proinflammatory neuropeptides like substance P and CGRP [82, 100]. As stated before, released ATP might stimulate these sensory nerve endings to enhance neurogenic inflammation and to maintain an inflamed state in the eye after a noxious insult.

Circulating ATP, nucleotides, and dinucleotides released into the aqueous humor can also stimulate purinergic receptors present in the trabecular meshwork, a tissue located at the iridocorneal angle of the anterior chamber of the eye and involved in the regulation of aqueous humor outflow. mRNA, protein, and functional evidence have been found for purinergic receptors P2Y_1_, P2Y_2_, P2Y_4_, and P2Y_11_ in the bovine trabecular meshwork (Figure 1) [101, 102] and in the human HTM-3 cell line [103]. Depending on the purinergic receptor activated, an increase or decrease in aqueous humor outflow is found. In this sense, selective agonists of P2Y_1_ receptor increase the facility of aqueous humor outflow and have been proposed as possible drugs for ocular hypertension [102]. On the other hand, ocular inflammation/uveitis produces the opposite effect on outflow facility (decrease) and it has been proposed that ATP and other inflammatory mediators might be involved in this effect [101, 104–106].

## 4. Conclusions

The eye has evolved to curb intraocular inflammation protecting the delicate visual elements from damage that would be detrimental to visual acuity. This ability of the eye to limit and control immune responses is known as ocular immune privilege. However, the immune privilege can fail and inflammatory processes can occur. The nucleoside adenosine and nucleotides such as ATP are emerging as novel molecules related to ocular inflammatory diseases. To date, the anti-inflammatory effects of adenosine and their agonists CGS21680 and CF101 acting via A_2A_ and A_3_ adenosine receptors, respectively, have encouraged exploring their use for the treatment of inflammatory ophthalmic conditions such as ocular retinal pathologies and dry eye and clinical trials are being developed. In contrast to adenosine, the nucleotide ATP exhibits proinflammatory actions mediated by purinergic P2 receptors present in sensory nerve endings or in other eye locations. Altogether the effects of nucleotides and dinucleotides suggest the development of some of these compounds as therapeutic agents mainly based on the use of P2 receptor antagonists. Also, indirectly, the use of P2Y_2_ agonists on the ocular surface to treat dry eye could reduce ocular surface inflammation, but it is necessary to be aware that the anti-inflammatory effect is a consequence of the restorage of aqueous and mucin production. Under these new normal conditions, friction of the lids with the ocular surface is diminished and therefore inflammation is reduced. In any case, to our knowledge, apart from the commented effects on dry eye, there is a lack of patents claiming the use of agonists or antagonists for the treatment of ocular inflammation, although, in the recent years, our knowledge about the relation of these molecules with ocular inflammatory processes is increasing. However a better understanding of their exact contribution in the different ocular inflammatory diseases (dry eye, severe cicatrizing conjunctivitis, uveitis, and so forth) is an important step to reveal additional pathologic mechanisms and designing new therapies based on the use of purinergic agonists and antagonists.
